# ENMD-1198, a novel tubulin-binding agent reduces HIF-1alpha and STAT3 activity in human hepatocellular carcinoma(HCC) cells, and inhibits growth and vascularization *in vivo*

**DOI:** 10.1186/1471-2407-8-206

**Published:** 2008-07-23

**Authors:** Christian Moser, Sven A Lang, Akira Mori, Claus Hellerbrand, Hans J Schlitt, Edward K Geissler, William E Fogler, Oliver Stoeltzing

**Affiliations:** 1 Departments of Surgery and Surgical Oncology, Regensburg Medical Center, Regensburg, Germany; 2 Department of Internal Medicine I; University of Regensburg Medical Center, Regensburg, Germany; 3 EntreMed Inc., Rockville, MD, USA

## Abstract

**Background:**

Hepatocellular carcinoma (HCC) represents a highly vascularized tumor entity and the process of angiogenesis is essential for the growth of HCC. Importantly, the pro-angiogenic transcription factors HIF-1α and STAT3 have been implicated in HCC progression, thus representing interesting targets for molecular targeted therapy. We hypothesized that therapeutic inhibition of HIF-1α could be achieved by using a novel tubulin-binding agent (ENMD-1198). ENMD-1198 is an analog of 2-methoxyestradiol (2ME2) with antiproliferative and antiangiogenic activity.

**Methods:**

The human HCC cell lines HUH-7 and HepG2 were used for experiments. Effects of ENMD-1198 on constitutive and inducible (hypoxia, growth factors) activation of signaling cascades, including HIF-1α and STAT3, were investigated by Western blotting. Changes in VEGF expression were determined by real-time PCR. Effects of ENMD-1198 on cancer cell migration and invasion were evaluated in *in vitro*-assays. The growth-inhibitory effects of ENMD-1198 (200 mg/kg/day) were determined in a subcutaneous tumor model (HUH-7).

**Results:**

ENMD-1198 inhibited the phosphorylation of MAPK/Erk, PI-3K/Akt and FAK. Moreover, activation of HIF-1α and STAT3 was dramatically reduced by ENMD-1198, which resulted in lower VEGF mRNA expression (P < 0.05). In addition, tumor cell migratory and invasive properties were significantly inhibited (P < 0.05, for both). *In vivo*, treatment with ENMD-1198 led to a significant reduction in tumor growth, tumor vascularization, and numbers of proliferating tumor cells (P < 0.05 for all).

**Conclusion:**

The novel microtubule destabilizing agent ENMD-1198 is suitable for inhibiting HIF-1α and STAT3 in human HCC cells and leads to reduced tumor growth and vascularization *in vivo*. Hence, inhibition of HIF-1α and STAT3 could prove valuable for therapy of hepatocellular carcinoma.

## Background

Hepatocellular carcinoma (HCC) is the fifth most cancer worldwide, with a continuously increasing incidence [1]. Importantly, therapy of patients with HCC remains challenging, as this tumor entity is highly resistant to systemic therapies, and only few patients qualify for surgical or ablative strategies due to advanced tumor stage or limited liver function. In addition, the post-interventional relapse rates for HCC are high, thus overall demanding the development of novel, i.e. molecular targeted, treatment strategies for improving outcome of patients with HCC.

In general, HCC represents a hypervascularized tumor and its progression is closely related to angiogenesis [2,3]. Recent studies have not only identified the vascular endothelial growth factor (VEGF) to be overexpressed in hepatocellular carcinoma, but also that the transcription factor HIF-1α plays a central role in HCC progression and angiogenesis [4-6]. Furthermore, the transcription factor signal transducer and activator of transcription 3 (STAT3), yet another inducer of angiogenesis in terms of up-regulating VEGF, is constitutively activated in HCC [7-9]. STAT3 has therefore drawn attention as a novel target for cancer therapy [8,10-12]. However, development of specific inhibitors to either HIF-1α, or STAT3 has proven difficult and research is ongoing. Nevertheless, certain compounds have lately been identified that exert an indirect anti-HIF-1α activity, such as 2-methoxyestradiol (2ME2) [13]. 2ME2 has been demonstrated to impair activation of HIF-1α through destabilization of microtubules, in addition to exhibiting antiproliferative and pro-apoptotic effects [13-15]. Moreover, 2ME2 has elicited growth-inhibitory and antiangiogenic properties in preclinical models of cancer [13,15-21] and clinical trials evaluating 2ME2 have been conducted [22-24]. However, the suitability of microtubule destabilizing agents for targeting HIF-1α in HCC has not been investigated to date.

The novel tubulin-binding compound ENMD-1198 (2-methoxyestra-1, 3, 5, (10) 16-tetraene-3-carboxamide) is a new chemical entity based on a modified chemical structure of 2-methoxyestradiol, which has been designed to improve the pharmacokinetic properties, growth-inhibitory, and antiangiogenic properties of 2ME2 [25]. Preclinical studies identified ENMD-1198 as an orally active, microtubule disrupting agent that leads to arrest of cell division and apoptosis in tumor cells. Recently, ENMD-1198 has entered a clinical phase I trial to evaluate the safety, tolerability, pharmacokinetics, and clinical benefit in patients with advanced cancer whose disease has failed to respond to existing therapies.

In the current study we hypothesized that ENMD-1198 could be used to inhibit HIF-1α activation in human hepatocellular cancer cells, which would reduce tumor growth and angiogenesis *in vivo*. Importantly, since estrogen receptors (ER) are known to be present in advanced HCC, the therapeutic use of an estradiol-analogons, such as 2ME2, for antineoplastic/antiangiogenic therapy is unknown [26-28]. Thus far, randomized controlled trials comparing anti-estrogen therapy with conservative treatment were discouraging and showed neither an antitumoral nor any survival benefit [27,29,30]. Importantly, ENMD-1198 is devoid of such estrogenic effects and therefore could offer new perspectives for therapy [25]. We therefore sought to investigate, whether ENMD-1198 is efficacious for treatment of HCC, with a particular focus on its anti-HIF-1α and antiangiogenic potential.

## Methods

### Cells and culture conditions

The human hepatocellular carcinoma cell lines HUH-7 and HepG2 were obtained from the American Type Culture Collection (ATCC, Manassas, VA). Cells were cultured in Dulbecco's modified Eagle's Medium (DMEM) or RPMI1640 (Gibco, Karlsruhe, Germany) supplemented with 10% fetal calf serum (FCS), and were maintained in 5% CO_2 _at 37°C. All *in vitro *experiments were done at 60% to 70% cell density. For *in vivo *experiments, trypsinized cells were resuspended in HBSS.

### Reagents and antibodies

ENMD-1198 was provided by EntreMed, Inc. (Rockville, MD, USA) [25]. For *in vitro *experiments, ENMD-1198 was dissolved in DMSO (0.1%) and equal concentrations of DMSO served as a control in all experiments. Antibodies against Akt, phosphorylated Akt^Ser473^, p44/42 MAPK1/2, phosphorylated p44/42 MAPK^Thr202/Tyr204^, signal transducer and activator of transcription 3 (STAT3), phosphorylated STAT3^Tyr705^, focal adhesion kinase (FAK) and phosphorylated FAK^Tyr925 ^were purchased from Cell Signaling Technologies (Beverly, MA). Anti-VEGF antibody was obtained from R&D Systems (Minneapolis, MN). Probing with an anti-β-actin antibody (Santa Cruz Biotechnology, Santa Cruz, CA) served as a loading control. Antibodies to HIF-1α (NB100–105) were purchased from Novus Biologicals (Littleton, CO). Recombinant human EGF and HGF were used in stimulation assays (R&D Systems).

### MTT analysis

To evaluate cytotoxic effects of ENMD-1198 on tumor cells, HUH-7 and HepG2 cells were seeded into 96-well plates (1 × 10^3^/well; 12 wells per condition) and exposed to various concentrations of ENMD-1198 for 24 and 48 hours at 37°C. We used the methylthiazole tetrazolium (MTT) assay to assess cell proliferation.

### Immunoblot analysis of constitutive and inducible signaling intermediates

To determine the effects of ENMD-1198 on signaling intermediates, cancer cells were incubated for 16 hours with FCS-RPMI containing ENMD-1198 (2,5 μM), prior to stimulation with recombinant human EGF (40 ng/ml, 5 min and 10 min; 1% FCS-RPMI). Whole cell lysates were prepared, as described elsewhere [31]. Protein samples (75 μg) were subjected to Western blotting on a denaturating 10% SDS-PAGE. Analyses for HIF-1α were performed on both nuclear protein extracts and whole cell lysates, as described [32]. In addition, tumor samples were subjected to Western blotting after lysis in RIPA-B buffer. [32,33]. Nuclear protein was extracted using a commercially available kit (NucBuster; Novagen, Merck Biosciences, Darmstadt, Germany). Experiments were performed in triplicates and confirmed in both cell lines (HUH-7, HepG2).

### Real-time PCR analyses

For real-time PCR (RT-PCR), total RNA was isolated using Trizol Reagent (Invitrogen, Karlsruhe, Germany) and subsequently purified by ethanol precipitation. For each RNA sample, a 1 μg aliquot was reversely transcribed into cDNA using the Superscript II Kit (Qiagen, Hilden, Germany). Primer pairs were as follows: VEGF165 (5'-GCACCCATGGCAGAAGGAGGAG; 3'-AGCCCCCGCATCGCATCAG), and ATF3 (5'-CTGCAGAAAGAGTCGGAG; 3'-TGAGCCCGGACAATACAC). Primers were optimized for MgCl2 and annealing, and PCR products were confirmed by gel electrophoresis. RT-PCR was performed using the LightCycler system and Roche Fast-Start Light Cycler-Master Hybridisation Probes master mix (Roche Diagnostics, Mannheim, Germany) [33]. Changes in VEGF-A expression were determined after 24 and 48 hours exposure to ENMD-1198 (2.5 μM).

### Enzyme-linked immunosorbent assay for VEGF protein

To determine changes in VEGF secretion by tumor cells, we used an ELISA kit specific to human VEGF-A (BioSource Europe, Nivelles, Belgium). HCC cells were plated at 40–50% density and incubated ± ENMD-1198 (2.5 μM, 20 hours). Analyses of culture supernatants were performed according to the manufacturer's protocol.

### Migration and invasion assays

To determine the effect of ENMD-1198 on cancer cell motility, migration assays were performed using modified Boyden chambers [32]. Briefly, 1 × 10^5 ^cells were resuspended in 1% FCS-RPMI and seeded into inserts with 8 μm filter pores, which were either uncoated (migration assay), or matrigel-coated (invasion assay) (Becton Dickinson Bioscience, Heidelberg, Germany). 1% FCS-DMEM ± EGF (40 ng/ml), or HGF (50 ng/ml), served as a chemoattractant. After 48 hours, cells were fixed and stained (Diff-Quick reagent, Dade Behring, Newark, NJ) and counted in four random fields.

### Animal models

Eight-week-old male athymic nude mice (BALB/c nu/nu) (Charles River, Germany) were used for experiments, as approved by the Institutional Animal Care and Use Committee of the University of Regensburg and the regional authorities. In addition, experiments were conducted according to *Guidelines for the Welfare of Animals in Experimental Neoplasia *published by The United Kingdom Coordinating Committee on Cancer Research. The effects ENMD-1198 on the growth of human hepatocellular carcinoma cells were evaluated in a subcutaneous xenograft model. In brief, 1 × 10^6 ^human HCC cells (HUH-7) were injected into the subcutis of nude mice. After implantation, tumors were allowed to grow 7 days (volume ~100 mm^3^) before treatment was initiated and mice randomized into one of two groups (n = 10/group) receiving either vehicle or ENMD 1198 (200 mg/kg/day) per oral gavage. Tumor diameters were measured every other day, and tumor volumes were calculated (width^2 ^× length × 0.5).

### Immunohistochemical analyses of tumor vascularization and cancer cell proliferation

Multiple cryo-sections and paraffin-embedded section were obtained from tumors for all immunohistochemical analyses. CD31-positive vessel area was assessed using rat anti-mouse CD31/PECAM-1 antibody (Pharmingen, San Diego, CA) and peroxidase-conjugated goat anti-rat IgG (Jackson Research Laboratories, West Grove, PA) on frozen sections, as previously described [32]. Antibody binding was visualized using stable diaminobenzidine. Images were obtained in four different quadrants of each tumor section (2 mm inside the tumor-normal tissue interface) at 40× magnification. Measurement of vessel area of CD31 stained vessels was performed by converting images to grayscale and setting a consistent threshold for all slides using ImageJ software (version 1.33; National Institute of Health, Bethesda, MD). Vessel areas were expressed as pixels per high-power field (HPF) [32]. To determine the amount of proliferating tumor cells, mice received intraperitoneal injections of BrdUrd (Sigma Aldrich, Germany) (1 mg/mouse) two hours prior to termination of animal studies. A commercially available BrdUrd-detection kit (Becton Dickinson) was used to visualize BrdUrd-uptake of cells in sections of tumors. Briefly, sections were incubated with anti-BrdUrd antibody solution, followed by streptavidin conjugated HRP-linked goat anti-mouse IgG2. Antibody binding was visualized by incubating slides in diaminobenzidine, with the aid of hematoxylin counterstaining. BrdUrd-positive tumor cells were counted in four fields per tumor section at 20× magnification and averages were calculated.

### Statistical analysis

Results of *in vivo *experiments were analyzed for significant outliers using the Grubb's test for detecting outliers . Tumor-associated variables in *in vivo *experiments were tested for statistical significance using the Mann-Whitney U test for non-parametric data. The two-sided student's t-test was applied for analysis of *in vitro *data. All results are expressed as the mean ± SEM.

## Results

### Effects of ENMD-1198 on growth of HCC cells *in vitro*

**Figure 1:**
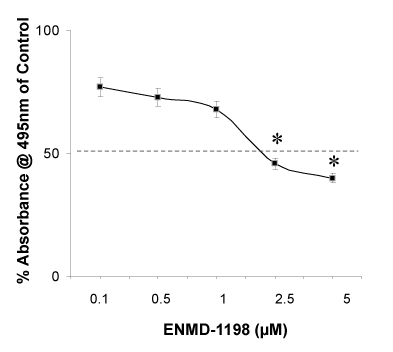
**Antiproliferative effects of ENMD-1198 on HCC cells**. HUH-7 cells were incubated for 24 h with various concentrations of ENMD-1198 and MTT reading was performed thereafter. ENMD-1198 dose-dependently elicited significant antiproliferative effects on HCC cells with an IC_50_at 2.5 μM in both cell lines (data shown for HUH-7) (*P < 0.05). Bars represent mean ± SEM from three independent experiments. Bars: SEM.

To determine potential cytotoxic and anti-proliferative effects of ENMD-1198, the human hepatocellular carcinoma cell lines HUH-7 and HepG2 were exposed to ENMD-1198 at various concentrations (0–5 μmol/L) and cell viability was determined by MTT analysis. Results show, that treatment with ENMD-1198 led to a significant dose-dependent inhibition of HCC cell growth *in vitro *(Fig. 1). The IC_50 _(24 hours) of ENMD-1198 was seen at 2.5 μmol/L (both cell lines) and this dose was used for all subsequent *in vitro *experiments. However, this IC_50 _appears to be higher compared to other cell cancer cell lines [25]. We conclude from these experiments that ENMD-1198 elicits anti-neoplastic activity on HCC cells, suggesting that this compound could be valuable for reducing growth of HCC *in vivo*.

### Effects of ENMD-1198 on cell signaling and migration of HCC cells

**Figure 2:**
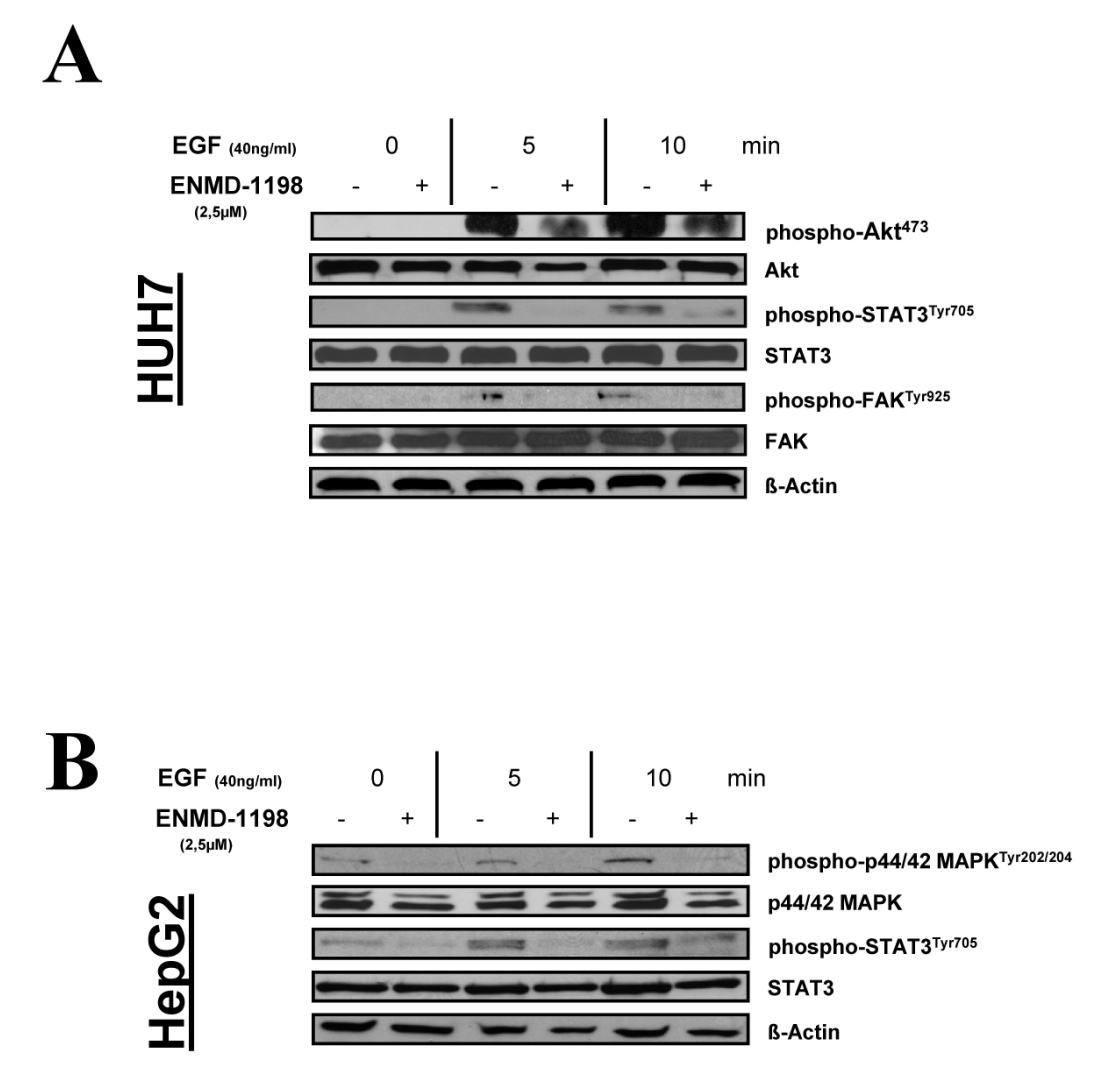
**Effect of ENMD-1198 on signaling in HCC cells**. Western blot analysis was performed to determine signaling pathways affected by therapy with ENMD-1198. Recombinant EGF (40 ng/ml) was used for stimulating cells after pre-incubation with ENMD-1198 (16 hours). A) HUH-7 cells and B) HepG2 cells were used for experiments. ENMD-1198 abrogated EGF-induced phosphorylation of Akt (HUH-7), FAK (HUH-7), p44/42 MAPK (HepG2), and STAT3 (HUH-7, HepG2). β-actin served as a loading control.

**Figure 3:**
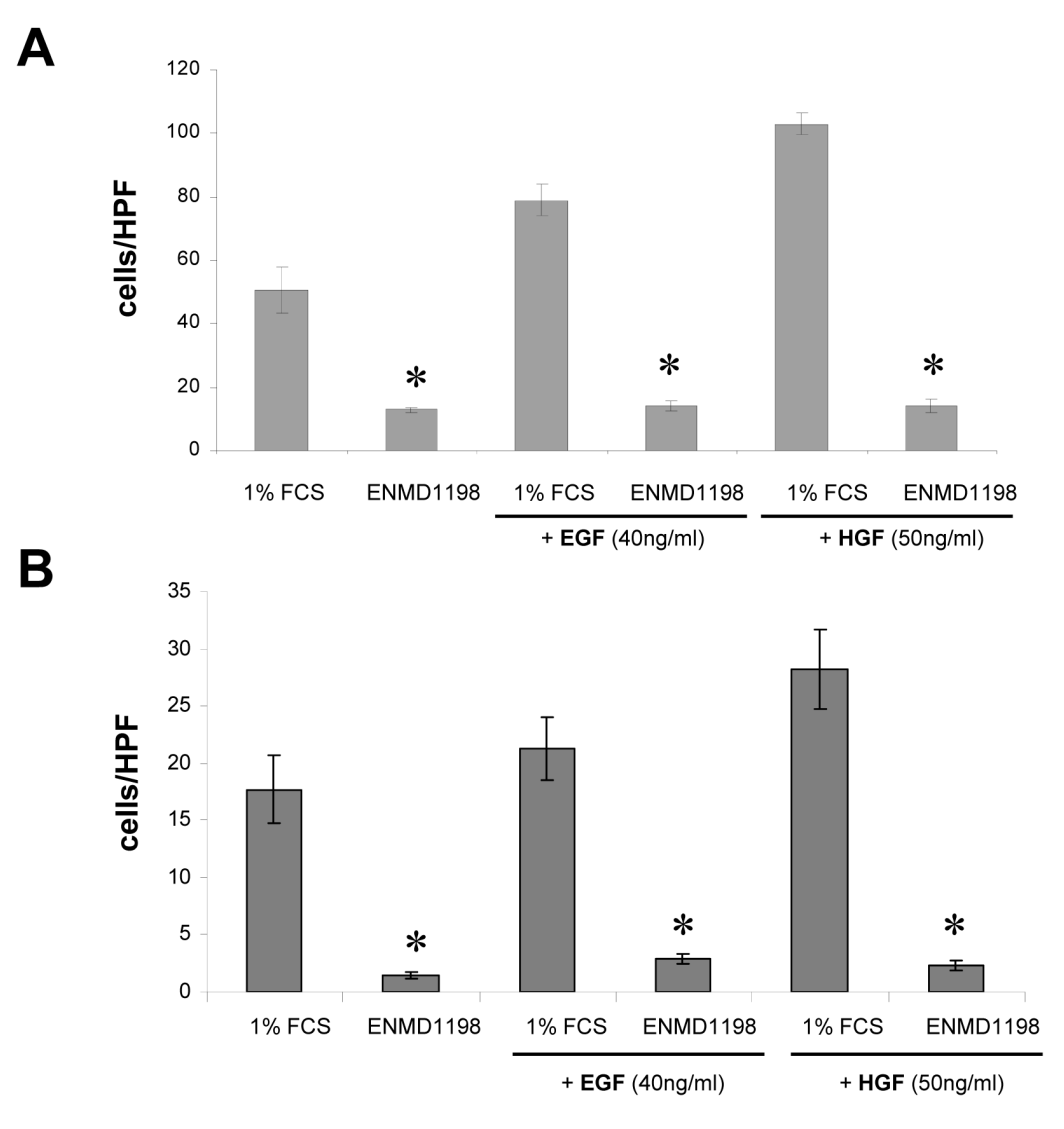
**Impact of ENMD-1198 on cancer cell migration and invasion**. The effects of ENMD-1198 on cancer cell migration were determined by using modified Boyden chambers. A) In migration assays (HUH-7), ENMD-1198 (2.5 μM) effectively reduced cancer cell motility and blunted the response to either EGF, or HGF (*P < 0.01). B) The effects on cancer cell invasiveness were evaluated using Matrigel-coated inserts. ENMD-1198 (2.5 μM) significantly abrogated EGF- and HGF-mediated invasive properties of hepatocellular carcinoma cells, compared to controls (*P < 0.01 for all). Experiments were performed in triplicates and results were confirmed in a second cell line (HepG2). Bars: SEM.

Since EGF is known to promote invasiveness and angiogenesis of HCC [34], we first investigated whether ENMD-1198 could be used for interfering with activation of pathways involved in EGF-signaling in HCC cells. Treatment with ENMD-1198 substantially disrupted EGF signaling in terms of diminishing downstream phosphorylation of the substrates p44/42 MAPK and Akt (Fig. 2A). Moreover, the activation of STAT3, an important transcription factor for regulating VEGF-A in cancer cells, was markedly reduced by ENMD-1198 (Fig. 2A, B). In addition, an EGF-induced phosphorylation of FAK, yet another essential mediator for cancer cell invasiveness, was markedly inhibited by ENMD-1198 (Fig. 2A). As a functional consequence of multiple signaling pathway inhibition and interference with transcriptional regulation, ENMD-1198 significantly inhibited both EGF- and HGF-mediated cancer cell migration and invasiveness (Fig. 3A, B). These experiments suggest that ENMD-1198 may effectively interfere with crucial EGF-mediated and angiogenesis-related signaling cascades in hepatocellular carcinoma cells. Hence, treatment with ENMD-1198 may not only affect tumor angiogenesis, but also harbors the potential to reduce metastasis through diminishing migratory and invasive properties of HCC cells.

### Effects of ENMD-1198 on nuclear HIF-1α expression and VEGF expression

**Figure 4:**
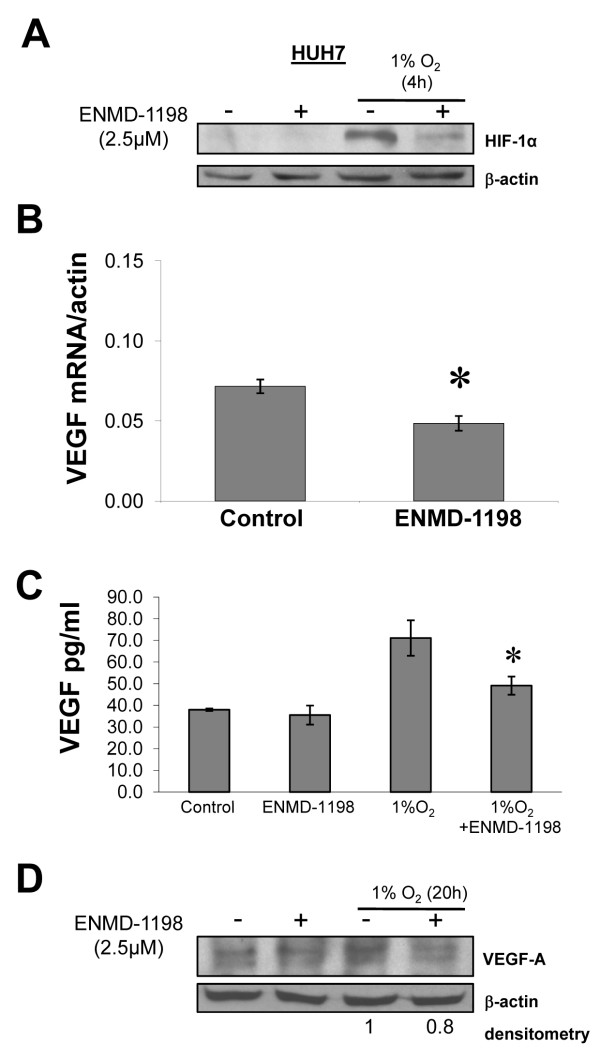
**Effect of ENMD-1198 on HIF-1α and VEGF expression in HCC cells**. To determine effects of ENMD-1198 on nuclear HIF-1α expression, HUH-7 cells were incubated for 16 h ± ENMD-1198 and subsequently exposed to hypoxia (1% O_2_, 4 h). A) Western blot analysis of whole protein showed that ENMD-1198 effectively blunted hypoxic induction of HIF-1α protein. B) In addition, treatment with ENMD-1198 down-regulated constitutive VEGF-A mRNA levels in HCC cells (HepG2), as measured by real-time PCR (* P < 0.01) (n = 3/group). VEGF-A mRNA expression is normalized to β-actin. C) ELISA analysis for VEGF in culture supernatants (HepG2). Hypoxia (20 h, 1% O_2_) markedly increased VEGF protein. ENMD-1198 treatment lowered VEGF secretion under hypoxic conditions (*P < 0.05). D) Western blot analysis for VEGF. Cells were incubated under either non-hypoxic, or hypoxic conditions ± ENMD-1198. Treatment with ENMD-1198 slightly lowered the hypoxic induction of VEGF, as determined by densitometry. Bars: SEM.

The transcription factor HIF-1α has been identified as an important promoter of HCC growth and angiogenesis [4,35]. Importantly, the ability of ENMD-1198 and 2ME2 to inhibit HIF-1α has recently been suggested in preclinical tumor models of solid malignancies [13,14]. We therefore investigated whether ENMD-1198 could also interfere with hypoxia-induced signaling in human hepatocellular carcinoma cells. Western blotting showed that treatment with ENMD-1198 markedly diminished a hypoxia-induced (1% O_2_) activation of HIF-1α in cancer cells (Fig. 4A). As HIF-1α is an important regulator of the pro-angiogenic molecule VEGF [36], we next determined the effects of ENMD-1198 on VEGF-A expression in HCC cells by real time PCR. Results show that treatment with ENMD-1198 slightly reduced constitutive (non-hypoxic) VEGF-A mRNA expression levels in HCC cells, though results were statistically significant (Fig. 4B). Similar modest effects of ENMD-1198 on VEGF protein were seen in an ELISA assay (Fig. 4C) and Western blot (Fig. 4D) under hypoxic conditions (20 h, 1% O_2_) *in vitro*. However, HCC cells in general expressed only low levels of VEGF in *in vitro *conditions, suggesting that other angiogenic factors may also be involved in tumor angiogenesis of these tumor cells when growing *in vivo*. We conclude from these experiments that treatment with ENMD-1198 effectively disrupts hypoxic signaling in HCC cells which is mediated through HIF-1α.

### Effects of ENMD-1198 on growth and vascularization of hepatocellular carcinoma *in vivo*

**Figure 5:**
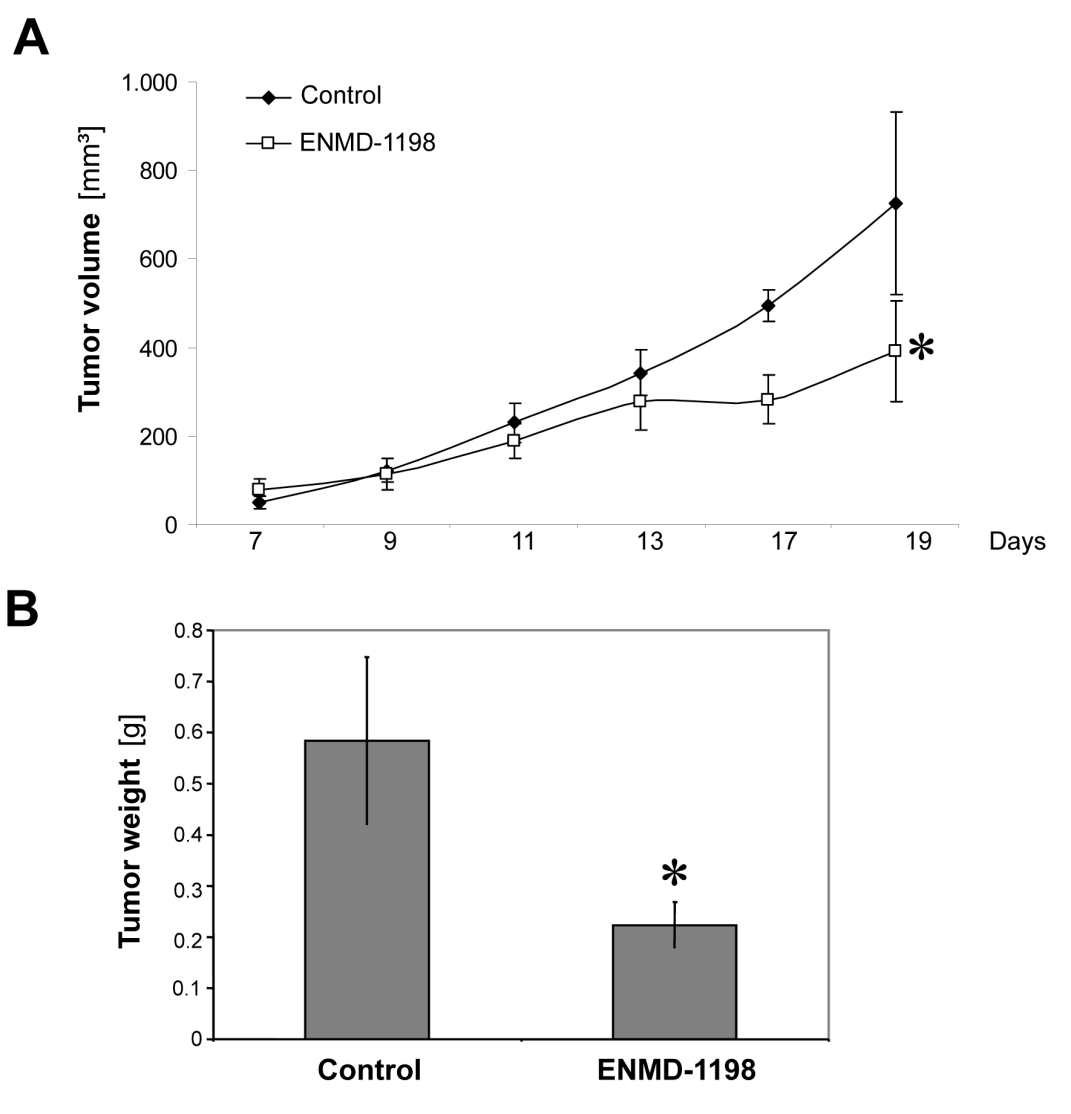
**Effect of ENMD-1198 on growth of colorectal cancer cells *in vivo***. To determine growth-inhibitory effects of ENMD-1198 *in vivo*, HUH-7 cells were implanted subcutaneously into mice (n = 10/group) which received either ENMD-1198 (200 mg/kg/day) or diluent when tumors became palpable. A) Treatment with ENMD-1198 led to a significant growth inhibition of xenografted hepatocellular tumors, compared to controls (*P < 0.05). B) Final tumor weights (day 19) in the ENMD-1198 treatment group were significantly lower, compared to excised tumors of the control group (*P < 0.05). Bars: mean ± SEM.

**Figure 6:**
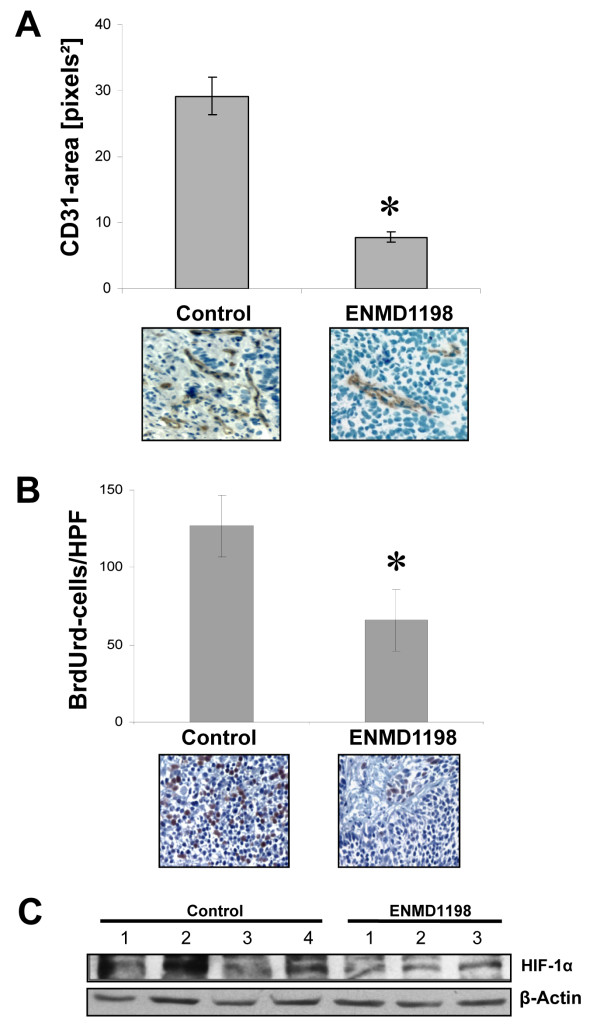
**Effect of ENMD-1198 on tumor vascularization and tumor cell proliferation**. A) Immunohistochemical analysis of tumor vascularization (CD31-positive vessels). Densitometric analysis of images from CD31-stained tissue sections from all tumors showed that vessel area was significantly reduced in ENMD-1198-treated tumors (*P < 0.01). B) ENMD-1198 significantly decreased the number of proliferating (BrdUrd-positive) tumor cells in tissue sections (*P < 0.01). For both vessel area and cell proliferation, representative images are illustrated. Results shown are the mean ± SEM. C) Western blot analysis for HIF-1α expression in HCC tumors showed a substantial reduction of HIF-1α in ENMD-1198 treated tumors.

To estimate growth inhibitory and antiangiogenic effects of ENMD-1198 *in vivo*, we used a subcutaneous tumor model (HUH-7 cells). Treatment with ENMD-1198 (200 mg/kg/day) significantly reduced growth of HUH-7 tumors, compared to controls (Fig. 5A). This reduction in tumor growth was also reflected by final tumor weights of excised tumors on day 19, which were significantly lower in the ENMD-1198 treatment group (Fig. 5B). Importantly, mouse body weights did statistically not differ among these two groups (controls: 22.1 ± 0.7; ENMD-1198: 20.3 ± 0.9 [mean ± SEM]; P = 0.15). Furthermore, vascularization of HUH-7 tumors in terms of CD31-positive vessel area was significantly reduced in tumor sections of the ENMD-1198 group (Fig. 6A). Moreover, treatment with ENMD-1198 also reduced the number of proliferating (BrdUrd-positive) tumor cells (Fig. 6B). Importantly, Western blotting of tumor tissues showed markedly decreased HIF-1α expression in ENMD-1198 treated tumors, suggesting that this target is inhibited *in vivo *(Fig. 6C). In conclusion, these experiments demonstrate that ENMD-1198 effectively inhibits growth of hepatocellular carcinoma through direct effects on the tumor cells, and also through inhibition of angiogenesis.

## Discussion

The present study shows that the 2ME2-analog ENMD-1198 interferes with growth factor-induced signaling in hepatocellular carcinoma and effectively inhibits HIF-1α and STAT3 activation. This disruption in cell signaling leads to a significant reduction of migratory and invasive properties of cancer cells and diminishes VEGF mRNA expression.

Tissue hypoxia constitutes as one of the main characteristics of the tumor microenvironment and has been shown to be implicated in the progression and metastasis of various solid malignancies [37-39]. One key regulatory protein in the cell response to changing oxygen levels is the hypoxia inducible factor-1α (HIF-1α). Interestingly, HIF-1α activation appears to be a very early event in carcinogenesis and this protein is expressed before histological evidence of angiogenesis or invasion even occur [40]. In regards to HCC, overexpression of HIF-1α has been reported, which has been associated with a poor prognosis [41-43]. Recently, both HIF-1α and VEGF were identified to be involved in the malignant transformation of dysplastic liver nodules and additionally a hypoxia-independent overexpression of HIF-1α has been shown to be involved in a model of mouse hepatocarcinogenesis [4,35]. Overall, these studies suggest that anti-HIF-1α therapy could prove valuable for treatment of HCC, or its pre-malignant lesions. However, specific inhibitors to HIF-1α are still under development, thus demanding the evaluation of alternative strategies for inhibiting HIF-1α in cancers. Interestingly, recent studies have demonstrated that 2ME2, an endogenous metabolite of estrogen, elicits antineoplastic efficacy which in part is mediated by an anti-HIF-1α activity [13,14]. The substance used in our study, ENMD-1198, is based on a modified chemical structure of 2ME2. However, ENMD-1198 has previously been evaluated in models of HCC. Indeed, we now demonstrate that ENMD-1198 effectively inhibits activation of HIF-1α in HCC, ultimately leading to a reduction of tumor growth and vascularization *in vivo*. However, the exact mechanism by which 2ME2 and ENMD-1198 inhibit HIF-1α is not fully understood. Recent studies suggested that 2ME2 treatment induces superoxide radicals in tumors [44,45], which in turn could down-regulate HIF-1α. Another potential mechanism for reducing HIF-1α activity could be mediated through interference with STAT3 phosphorylation, as STAT3 is required for forming an active HIF-1 complex [46]. In HCC, STAT3 appears to play a central role, as this transcription factor is implicated in oncogenesis and metastasis [7,9,12]. Furthermore, STAT3 appears to be necessary for a PI-3K/Akt-mediated hypoxia-independent HIF-1α protein synthesis [46,47]. Importantly, our results indicate that ENMD-1198 effectively diminishes phosphorylation of STAT3 in HCC cells, a finding which has not been reported for 2ME2 compounds to date. Hence, we now provide evidence for another mechanism for impairing HIF-1α function in cancer cells by 2ME2-like inhibitors in terms of diminishing STAT3 activation. Moreover, ENMD-1198 also reduced phosphorylation of Akt, a signaling intermediate that is up-stream of HIF-1α, thus resulting in further impairment of HIF-1α activity. This was also reflected in Western blot results for HIF-1α expression analysis in tumor tissues, which were markedly reduced in the ENMD-1198 treated tumors. In fact, one has to realize that tumor sizes differ significantly among these treatment groups, which per se could affect HIF-1α expression. Nevertheless, we also detected a diminished STAT3 activation by Western blotting of tumor tissues in the ENMD-1198 treatment group (data not shown), suggesting that the drug also effectively inhibits both targets *in vivo*.

Another important aspect is the observed anti-metastatic property of ENMD-1198 *in vitro*, as treatment of HCC cells led to reduced phosphorylation of the pro-migratory signaling component FAK. In *in vitro *assays, ENMD-1198 substantially abrogated EGF- and HGF-mediated cancer cell migration and invasiveness, suggesting that this substance could be valuable for reducing HCC metastasis. Development of intra-hepatic metastases is a common problem in HCC patients who underwent liver resection for HCC, or are on the waiting list for transplantation. Since HIF-1α and STAT3 both promote metastasis formation, treatment with ENMD-1198 appears to be reasonable and warrants further investigation. ENMD-1198 is already being investigated in a phase I trial in advanced cancers and results from these trials will be important for further defining the suitability of this agent in cancer therapy.

## Conclusion

In conclusion, our study demonstrates that treatment with ENMD-1198 harbors significant potential for reducing growth and vascularization of hepatocellular carcinoma. Importantly, ENMD-1198 effectively diminishes both HIF-1α and STAT3 activation in HCC cells, which represent important mediators of HCC progression. Our data are therefore in favor of evaluating this concept of targeting microtubules in clinical trials for therapy of human hepatocellular carcinoma.

## Competing interests

The authors CM, SAL, AM, CH, HJS, EKG and OS have no competing interest to declare. The co-author WEF is employee at EntreMed Inc., Rockville MD.

## Authors' contributions

The authors CM and SAL performed the *in vitro *and *in vivo *experiments, analyzed the data and prepared the manuscript, AM performed staining procedures, CH assisted in experimental design and manuscript review, HJS and EKG edited and reviewed the manuscript, WEF provided the substance, technical information and reviewed the manuscript, OS is principal investigator, designed the experimental outlines, aided in manuscript preparation, reviewed and revised the manuscript. All authors read and approved the final manuscript

## Pre-publication history

The pre-publication history for this paper can be accessed here:


